# Titania Enhanced Photocatalysis and Dye Giant Absorption in Nanoporous 1D Bragg Microcavities

**DOI:** 10.1021/acsanm.2c00477

**Published:** 2022-04-07

**Authors:** Victor J. Rico, Halime Turk, Francisco Yubero, Agustin R. Gonzalez-Elipe

**Affiliations:** Instituto de Ciencia de Materiales de Sevilla (CSIC-Univ. Sevilla), Avda. Américo Vespucio 49, E-41092 Sevilla, Spain

**Keywords:** TiO_2_ photocatalysis, nanocolumnar multilayer, nanoporous 1D Bragg microcavity, light trapping, dye giant absorption, visible light photodegradation

## Abstract

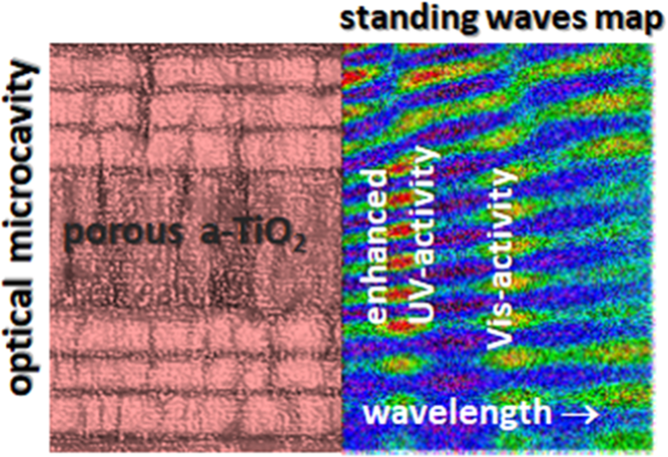

Light trapping effects are known to boost the photocatalytic degradation of organic molecules in 3D photonic structures of anatase titania (a-TiO_2_) with an inverse opal configuration. In the present work, we show that photocatalytic activity can also be enhanced in a-TiO_2_ thin films if they are incorporated within a nanoporous 1D optical resonant microcavity. We have designed and manufactured multilayer systems that, presenting a high open porosity to enable a straightforward diffusion of photodegradable molecules, provide light confinement effects at wavelengths around the absorption edge of photoactive a-TiO_2_. In brief, we have observed that a nanoporous 1D Bragg microcavity prepared by electron beam evaporation at oblique angles comprising a central defect layer of nanoporous a-TiO_2_ boosts the photocatalytic degradation of nitrobenzene and methyl orange dye solutions. The multilayer structure of the microcavity was designed to ensure the appearance of optical resonances at the a-TiO_2_ layer location and wavelengths around the absorption onset of this semiconductor. In this porous 1D Bragg microcavity, the diffusion constraints of molecules through the capping layers covering the a-TiO_2_ are effectively compensated by an increase in the photocatalytic activity due to the light confinement phenomena. We also report that the absorption coefficient of methyl orange dye solution infiltrated within the pore structure of the microcavity is exalted at the wavelengths of the corresponding optical resonances. This effect gives rise to a small but non-negligible visible light photodegradation of dye molecules. The possibilities of tailoring the design of 1D photonic systems to boost the photocatalytic activity of a-TiO_2_ are discussed.

## 1 Introduction

The UV photocatalytic activity of anatase TiO_2_ (a-TiO_2_) has been a recurrent research topic during the last decades due to its effectiveness for pollution removal from gas or liquid sources.^1^ Anatase is the most popular photoactive phase of TiO_2_ mainly because it can be obtained by annealing treatments at relatively mild temperatures and is very stable under photoactivation conditions.^2^ In this context, much effort has been dedicated to increasing the reaction yields through careful manufacturing and control of the structural and microstructural characteristics of the active rutile and anatase phases of this semiconductor oxide.^3,4^ Among the strategies considered, we can refer to the doping with transition-metal cations,^5^ the preparation of defective titania,^6^ or the implementation of tandem catalyst concepts.^2^ Recently, a different successful approach in this regard has been the application of light confinement effects to increase the photocatalytic performance of the system.^7^ For this purpose, a-TiO_2_ has been fabricated either in the form of three-dimensional photonic crystals (3D-PCs), usually depicting an inverse opal structure,^8,9^ or as agglomerated particles or nanoparticles of a-TiO_2_ in contact with 3D-PCs made of titania or other materials (e.g., SiO_2_,^10^ SnO_2_,^11^ or TiO_2_^12^). A common feature in these 3D-PCs is the slow group velocity of light for certain wavelengths and spatial locations. For photons energies around the absorption edge of the semiconductor, or higher values, this effect enhances its absorption and, therefore, the photoactivity of the system.^13^ Tuning the spectral response in these systems is possible by adjusting the dimensions of the basic building blocks of the photonic structures.^14^ In the case of inverse opals, these building blocks correspond to the size of the empty spheres arranged according to a 3D ordered distribution.^15^ The combination of the light confinement effects appearing in these inverse opals with plasmonics,^16^ other semiconductors active in the visible,^17,18^ doping of the a-TiO_2_ to shift its absorption edge toward the visible region,^12^ or the promotion of its sensitization with dyes^19^ has opened new perspectives in the quest for advanced concepts in PC-assisted light-activated titania photocatalysis.^20^

Photonic crystals can be made one-dimensional (1D-PCs), one example of which corresponds to the so-called Bragg mirrors (BM) formed by the alternant stacking of layers of two dielectric materials with refractive index contrast.^21^ The transmittance of this type of multilayer depicts a photonic band gap whose spectral position and width depend on the characteristics (thickness and refractive index) of the stacked layers. A modification of this photonic structure through the incorporation of a central layer with different thickness and/or refractive index gives rise to a so-called Bragg microcavity, characterized by the appearance of optical resonances within the photonic gap.^22^ 1D-PCs have been prepared by wet^21,23^ or vacuum/plasma routes. The latter methods offer advantages in terms of scalability to large areas and compatibility with sensitive substrates.^24−26^

Light confinement effects in 1D-PCs have been successfully used to improve the efficiency of dye-sensitized solar cells.^27,28^ They have also claimed to be responsible for the enhancement of the absorption coefficient of dye solutions infiltrated within the pore structure of 1D Bragg microcavities (i.e., to induce a so-called “giant” absorption).^29^ However, to our knowledge, except for some attempts to stack TiO_2_ and graphene oxide layers,^30,31^ 1D-PC structures have not been utilized to enhance the photocatalytic activity of a-TiO_2_. The present work demonstrates that light trapping in nanoporous 1D Bragg microcavities effectively enhances the photoactivity of this semiconductor. To prove this concept, we have designed and prepared a series of highly porous a-TiO_2_-based layered systems and correlate their photoactivity with the existence of light confinement effects for wavelengths around the onset of absorption of a-TiO_2_ (i.e., ∼380 nm). These nanostructured multilayered systems have been manufactured by electron beam evaporation in an oblique angle configuration (OAD),^32,33^ a procedure known to render columnar thin films with empty volume ratios up to 50%. We and other laboratories have applied this procedure to the fabrication of single photocatalytic layers of TiO_2_ and other materials^34−36^ or the fabrication of dye-sensitized solar cells.^37,38^

Herein, we have investigated the photodegradation rates of nitrobenzene and methyl orange dye solutions in contact with various layered systems where a photoactive nanoporous a-TiO_2_ layer is in the form of a bare film (sample pA). It is sandwiched between two SiO_2_ nanoporous layers (S/pA/S) or between two nanoporous BMs, with a (LH)^3^L layered structure, forming a BM/pA/BM microcavity stack. The L and H layers correspond to low- and high-refractive-index materials made of photocatalytic inactive SiO_2_ and Ta_2_O_5_. The thickness of the nanoporous anatase titania layer was the same in all these structures. The purpose of the S/pA/S sample is to limit reactants and products in/out-diffusion up to the a-TiO_2_ layer in a similar manner to the BMs. Meanwhile, the BM/pA/BM stack depicts a 1D Bragg microcavity photonic configuration^26,29^ and has been designed in such a way that two of their optical resonances appear (i) in the UV region at the onset of a-TiO_2_ absorption and (ii) in the visible region around the maximum absorption of the methyl orange dye, always after liquid infiltration (either water or dye solutions) of the porous layered structures. Through the comparison of the photocatalytic activity of these systems, we have found that the dye degradation is enhanced in the BM/pA/BM stack, which is specifically designed to provide light trapping at the central nanoporous titania layer for photon energies around the absorption edge of this semiconductor.

A related feature in this porous microcavity infiltrated with methyl orange molecules has been the observation of an enhancement of the absorption coefficient of the dye at the wavelengths of the optical resonances, as expected for a “giant absorption effect” reported by us in a previous work.^29^ The detection of certain photocatalytic dye degradation activated with visible light in this microcavity suggests the contribution of a photodegradation mechanism involving a direct electron transition from an excited state of the dye molecule to the conduction band of TiO_2_, followed by its subsequent degradation by reaction with the medium.

## 2 Materials and Methods

### 2.1 Sample Preparation

**Figure 1:**
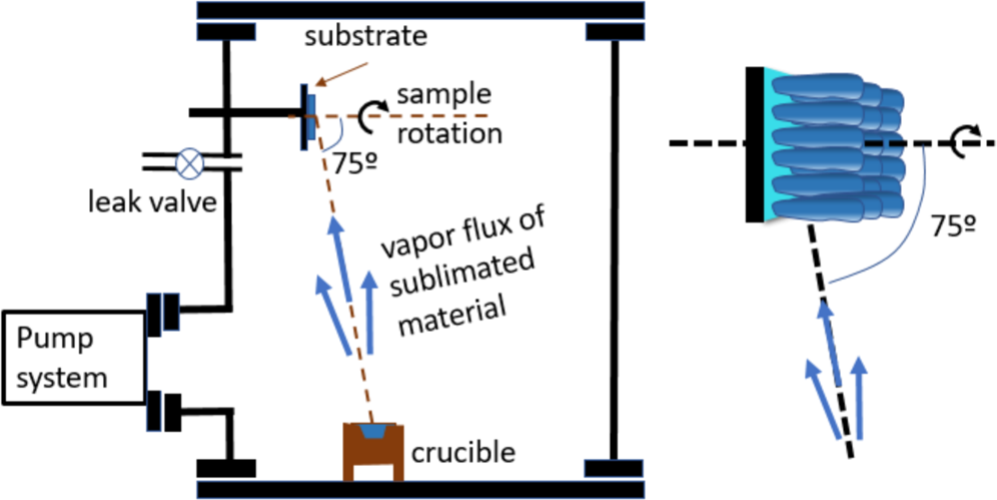
Schematic representation of the OAD setup and the typical nanocolumn structure formation induced by azimuthal sample rotation with this procedure.

Titania-based layered nanocolumnar structures were prepared by electron beam evaporation in an oblique angle deposition (OAD) geometry at a zenithal angle of 75° and a distance between target and substrates of 50 cm. Either fused silica plates or polished silicon wafers were used as substrates. TiO (i.e., titanium monoxide), SiO_2_, and Ta_2_O_5_ pellets (Kurt J. Lesker Company) were used as target materials for evaporation to produce TiO_2_, SiO_2_, and Ta_2_O_5_ thin films, respectively. A small oxygen leak of 3.0 × 10^–4^ mbar was dosed during the evaporation to ensure that the deposited layers grew in their fully oxidized stoichiometric forms. During deposition, the substrates were azimuthally rotated at a rate of 30 turns per minute. It is known that this rotation during OAD gives rise to a microstructure formed by vertical nanocolumns.^32,39^Figure 1 shows a schematic representation of the deposition setup and the typical nanocolumn structure formation induced with this procedure. More detailed information about the preparation and properties of single layers of these materials can be found in refs (36−38, 40−44). All samples were annealed at 400 °C in air for 3 h to induce the crystallization of the titania layers into the photocatalytically active anatase phase (a-TiO_2_) of this oxide.

Three different layered samples were fabricated with a similar amount (i.e., thickness) of photoactive a-TiO_2_: a bare porous a-TiO_2_ film (sample pA); a porous a-TiO_2_ film sandwiched between porous SiO_2_ layers, i.e., with a SiO_2_/a-TiO_2_/SiO_2_ trilayer structure (sample S/pA/S); and a porous 1D Bragg microcavity consisting of a-TiO_2_ film sandwiched between BMs (sample BM/pA/BM). The BMs had an (LH)^3^L multilayer structure, where L/H corresponds to low-/high-refractive-index materials (i.e., SiO_2_ and Ta_2_O_5_, respectively). Nanocolumnar thin films of these materials are photocatalytically inactive and absorb light at wavelengths shorter than 300 nm, i.e., they are transparent below 380 nm, the absorption onset of TiO_2_.

### 2.2 Structural, Microstructural, and Optical Characterization

X-ray diffraction (XRD) measurements were carried out in a Siemens D5000 diffractometer system, employing monochromatic Cu Kα radiation, 0.02° step angle, and 60 s measuring time per step. Microstructural characterization in a scanning electron microscopy (SEM) was carried out for thin films deposited on a silicon wafer that was diced for cross-section analysis. SEM images were taken with a Hitachi S4800 field emission electron microscope operated at 2.0 kV. UV–vis transmission spectra were recorded in a PerkinElmer spectrometer (UV/vis/NIR spectrometer Lambda 750S) for samples deposited on fused silica substrates. Spectra were recorded for pristine samples (i.e., with their pores empty) or for either water or aqueous dye solutions infiltrated samples (i.e., with their pores filled with these liquids).

### 2.3 Design of 1D Photonic Structure

The BM/pA/BM 1D Bragg microcavity was designed in such a way that when immersed in the aqueous dye solutions, it depicts optical resonances at the onset of the absorption edge of a-TiO_2_, (i.e., at about 380 nm) and around the maximum of the absorption band of methyl orange molecule (ca. 440–480 nm), one of the dyes considered in the photodegradation studies reported in this work. The overall thickness of the capping layers atop and beneath the a-TiO_2_ thin film (i.e., SiO_2_ layers in S/pA/S or the BM stack in BM/pA/BM microcavity) was about the same (400–500 nm) to induce similar diffusion restrictions to the infiltration of the reactant and product molecules, these latter resulting from the dye photodegradation process at the a-TiO_2_ middle layer. This ensures that any significant difference in photocatalytic activity between S/pA/S and BM/pA/BM samples responds exclusively to their different photonic performance (i.e., to the trapping effect of light with certain wavelengths within the microcavity) and not to diffusion constraints.

The transmittance of the samples was modeled using the transfer matrix method with the WVASE software (J. A. Woollan Co.). The parameters obtained from the fitting procedure (thicknesses and refractive indices of all of the layers within the considered multilayer stack) were used to evaluate the spatial distribution of the electric field amplitude vs wavelength, corresponding to the standing waves developed at the photonic multilayers. For this purpose, FilmStar Optical Thin Film software was used.^45^

### 2.4 Evaluation of Photocatalytic Activity

The photocatalytic activity of the nanoporous a-TiO_2_-based samples described above was evaluated following the evolution vs time of the absorbance of nitrobenzene NB (1.0 × 10^–4^ M) and methyl orange MO (1.7 × 10^–5^ M) aqueous dye solutions under UV irradiation. Once prepared, these solutions were stable under ambient illumination even after a prolonged contact with the layered samples. The experimental setup schematized in the Supporting information (SI), Figure S1, was used for these experiments. A fused silica cuvette (2 × 1 × 4 cm^3^) was filled with 4 cc of dye solution. The photoactive samples (2 × 2 cm^2^) deposited on a polished silicon wafer substrate were immersed in the dye solution filling the cuvette. A small flow of oxygen was continuously bubbled through the dye solution to ensure that the photodegradation kinetics was not limited by any shortage of this reactant. A Teflon cap with a small hole prevented the removal of liquid by the oxygen bubbles during the photodegradation experiment. Tests were carried out irradiating with a Xe lamp (LASING ASB-Xe-175) located at 15 cm from the cuvette, with an irradiance of 1.8 W cm^–2^ at the position of the samples for the complete UV–vis spectrum (ca. 0.3 W cm^–2^ corresponded to photons with a wavelength shorter than 380 nm). The kinetics of the photodegradation process was monitored following the time evolution of the maximum of the absorption bands of NB and MO dyes at, respectively, 268 and 466 nm. The absorbance spectra were recorded with two optical fibers provided with collimators lenses and located face to face at the two lateral windows of the cuvette; one of the fibers was used to illuminate and the other to collect the transmitted light, transversally to the irradiation with the Xe lamp used to activate the photodegradation of the dye solutions. An Ocean Optics “MAYA 2000 Pro” UV–vis spectrometer was used to collect absorbance spectra every 5 min for 2 h. It is assumed that the dye concentration in the solution is proportional to its absorbance. For some experiments, a PMMA plate was placed between the Xe lamp and the cuvette to act as a visible filter enabling the irradiation with just the photons of the visible spectrum emitted by the Xe lamp.

The selection of NB and MO dyes for the photodegradation experiments was motivated by their different molecular size (∼0.5 vs ∼1.2 nm, respectively) and the position of their absorption bands, in the far-UV region for the former and in the visible region for the latter. In principle, the bigger size of the MO molecule will impose more constraints for its diffusion through the pores of the capping multilayers. Another difference between these two molecules is that the NB solution is stable upon irradiation with the Xe lamp, while the MO solution underwent a little but progressive degradation upon UV irradiation, even in the absence of the photocatalyst. To account for this dye degradation induced by direct UV light exposure, the degradation kinetics data are reported normalized, i.e., in the form of *C*/*C*_0_ for the NB solution (with *C*_0_ the concentration of NB at irradiation time *t* = 0) or *C*/*C*_r_(*t*) for the MO solution (with *C*_r_(*t*) the concentration of the MO at irradiation time *t* upon exposure of the dye solution to the Xe lamp without photocatalytic agent). Two selected examples of the evolution of the absorption bands of NB and MO during photodegradation experiments under Xe lamp irradiation in the presence of the pA photocatalyst are shown in Supporting Information Figure S2. The intensities at the maxima of the corresponding absorption bands at 268 and 466 nm are used to characterize the photodegradation kinetics of the different nanoporous a-TiO_2_ samples, as explained in the previous paragraph.

## 3 Results and Discussion

### 3.1 Microstructure and Optical Characterization

Prior to any dye degradation study, samples were annealed in air at 400 °C for 3 h to induce the crystallization of the photoactive anatase phase of the titania layer.^1^ XRD spectra of these samples showing the formation of anatase titania can be seen in Supporting Information Figure S3. It is noteworthy that this annealing treatment did not affect the microstructural integrity of the multilayer stacks, which remained intact after the heating treatments.

**Figure 2:**
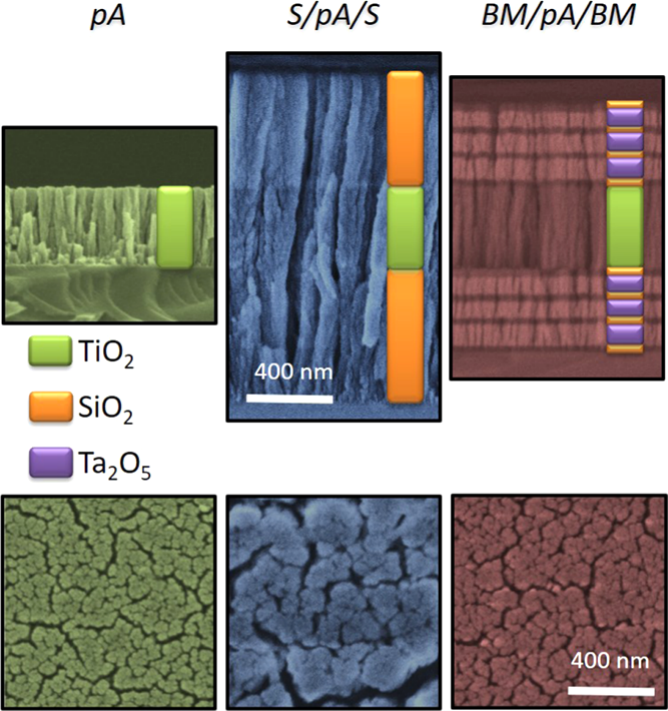
(Top) Cross-section and (bottom) front-view micrographs of, from left to right, a bare porous a-TiO_2_ layer (pA), a porous a-TiO_2_ layer sandwiched between two porous silica layers (S/pA/S), and a porous a-TiO_2_ layer sandwiched between two porous Bragg mirrors (BM/pA/BM). The colored schemes describe the material layer distribution. The brightness intensity of the individual layers in the cross-section micrographs agrees with their atomic composition.

The microstructure of the samples is depicted in Figure 2. It shows the cross-section and normal-view SEM backscattered electron micrographs of pA, S/pA/S, and BM/pA/BM samples. Colored schemes illustrate the distribution of the different material layers in the stacks. The SEM micrographs show that the three samples present the vertical nanocolumnar microstructure typical of OAD films and multilayers prepared under substrate azimuthal rotation.^31,39^ The brightness intensity of the individual layers observable in the cross-section micrographs in this figure agrees with their atomic composition. A characteristic of this microstructure is that the constituent nanocolumns, visible in the cross-section micrographs with a width of approximately 60–90 nm, are well separated by large void spaces forming a continuous nanoporous structure that communicates the sample surface with the interface with the substrate. Previous investigations with this type of samples have revealed that they have a void fraction of 40–60%, depending on experimental parameters of the deposition process.^32,42^

Another characteristic feature is that pores arrange along open channels of a rather large size (larger than 10 nm) and that quite small nanopores with a size less than or equal to 2 nm are present within the nanocolumns.^42,43^ We assume that this microstructure enables a straightforward diffusion of dissolved molecules or vapors, as proved in previous studies dealing with the photonic analysis of fluids and volatile organic compounds.^26,46,47^

It is also apparent in Figure 2 that the thickness of the a-TiO_2_ photoactive layer in the three samples is about the same (∼400 nm) and the thickness of the external SiO_2_ or SiO_2_/Ta_2_O_5_ capping stack in S/pA/S or BM/pA/BM sample ranges between 500 and 600 nm.

**Figure 3:**
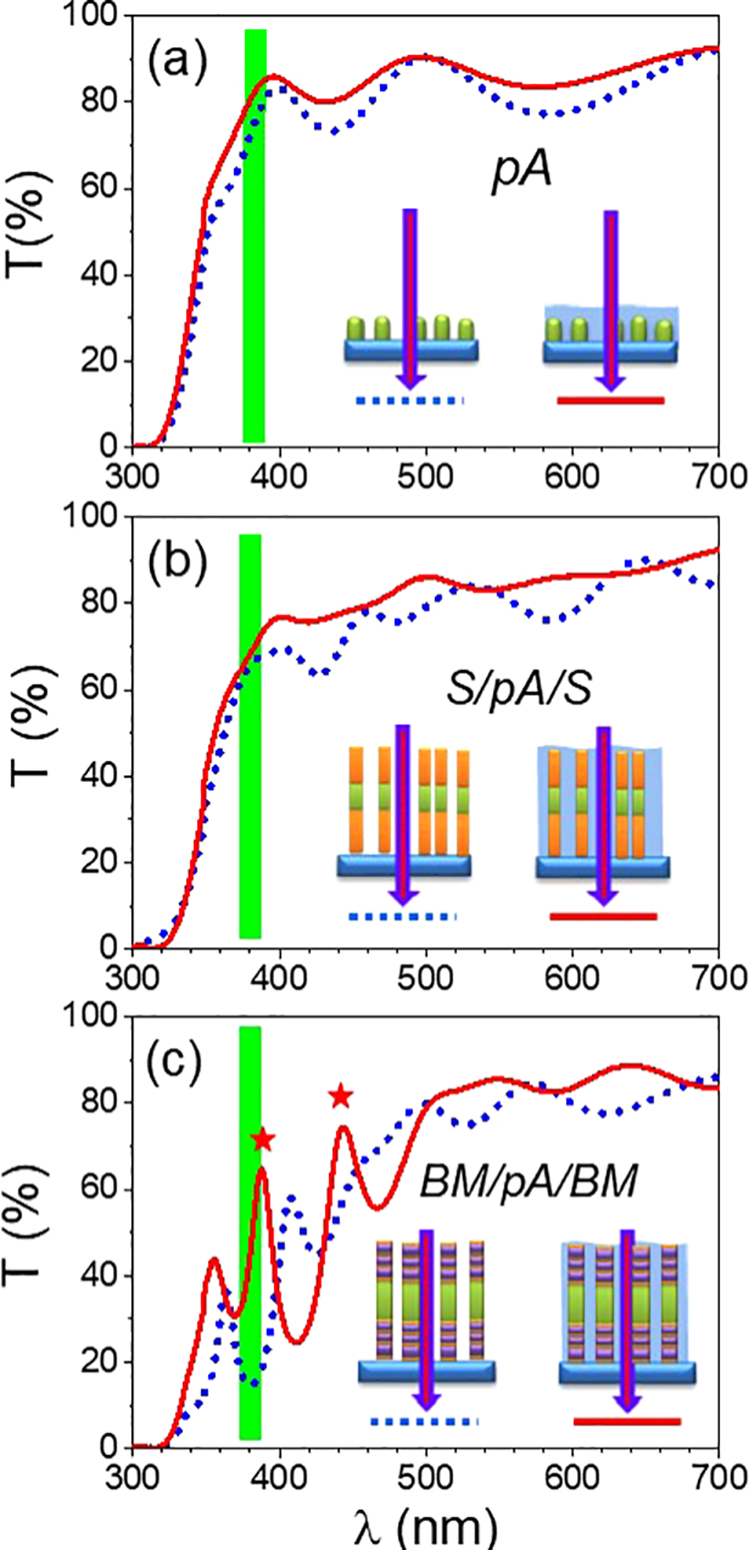
UV–vis transmission spectra recorded at normal incidence for anatase titania-based samples in their pristine state (dashed blue lines) or immersed in water (solid red lines): (a) pA, (b) S/pA/S, and (c) BM/pA/BM. The vertical broad green line indicates the onset of titania absorption. The stars denote the optical resonances in the water-infiltrated 1D Bragg microcavity. The schemes describe the pristine and water-infiltrated states of the three samples.

Figure 3a shows that the bare anatase layer (pA sample) depicts the typical transmittance of a titania thin film characterized by an absorption onset around 380 nm. Noteworthy, it is noteworthy the difference between the interference patterns of the spectra recorded for the pA sample either pristine or immersed in water. This pattern accounts for the refractive index (RI) contrast between the film material and the liquid medium surrounding the film/multilayers or infiltrated within their pores in each case, either air (RI = 1.00) or water (RI = 1.33). An evaluation of the RI of the bare a-TiO_2_ sample in each case using a conventional fitting analysis of the interference fringe and assuming a Cauchy-like wavelength dispersion^48^ rendered values of 1.83 and 1.93 at 450 nm for the “empty” and “water-filled” a-TiO_2_ layers, respectively. A rough estimate of porosity based on a Bruggeman effective medium approximation^49,50^ yields that approximately 40% of the volume of the a-TiO_2_ film is infiltrated with liquid.

At this point, it is worth mentioning that it is possible to enhance the void fraction of the layers by modifying the deposition conditions, especially using more glancing deposition geometries or lower deposition pressures.^26,32^ However, these possibilities have not been tested in this work because of the need to properly define the interfaces between layers, a feature that may be altered when excessively increasing the film porosity.

The transmittance spectra of sample S/pA/S (Figure 3b) are similar to those of sample pA, although the interference profile is weaker due to the optical interferences induced in the trilayer stack (the RIs of the pristine and water-infiltrated SiO_2_ layers prepared by OAD are around 1.26 and 1.38, respectively^43^).

**Figure 4:**
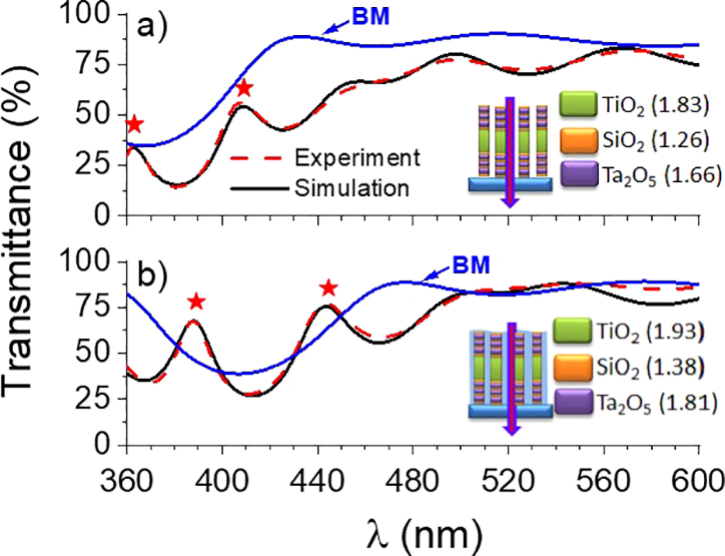
Experimental (red dashed lines) and simulated (solid black lines) transmittance spectra of sample BM/pA/BM deposited on a fused silica plate, in its pristine (i.e., pores empty) (a) or water-infiltrated (b) states. The solid blue lines are simulations of the (LH)^6^ Bragg mirror (BM) multilayer structure sandwiching the nanoporous a-TiO_2_ active layer in the microcavity. The stars indicate the optical resonances of the microcavity. The schemes describe the empty and filled states of the sample in each case (in parenthesis effective refractive indices of the corresponding layers).

According to the spectrum in Figure 3c, drastic changes occur in the transmittance of sample BM/pA/BM in comparison with those of pA and S/pA/S samples. The spectra correspond to the typical transmittance of a resonant microcavity with resonances within the gap of the BMs integrated in the microcavity. In addition, it is remarkable that filling the pore structure of sample BM/pA/BM with water induces a redshift of the spectral features. Of particular relevance is the fact that two optical resonances of this microcavity appear at around 385 and 440 nm after water flooding, a result that is deemed crucial to account for the enhancement of photocatalytic activity of this sample. As it is discussed below, this increase is attributed to light trapping phenomena within these 1D-PCs. The effect of water infiltration was simulated using the Bruggeman effective medium approximation. As a result of the fitting procedure, it was found that the pore volume of the microcavity was about 40% of total volume and that this porosity was completely filled with water through the infiltration process. Besides, according to the simulation analysis, the peaks at 385 and 440 nm appear to correspond to the 5th and 6th resonant modes of the microcavity, respectively (for further details of the fitting procedure and optical constant of the different layers in the microcavity, see Supporting Information S4, Tables S1 and S2). For illustration, a simulation of the transmittance spectra of this sample is reported in Figure 4, together with the spectra simulated for a BM structure (LH)^6^, which is the base of the resonant microcavity design. Note that the optical resonances of the microcavity lay within the gap of the Bragg mirror. It was also realized that the spectral shape in the near-UV region around the absorption edge of TiO_2_ can be accounted for by the convolution of an interference spectrum typical of a 1D Bragg microcavity and the corresponding absorption onset of anatase.

### 3.2 Light Confinement Effects in Resonant 1D Microcavities

The multilayer structure of the BM/pA/BM Bragg microcavity was designed in such a way that, when immersed in water, one of its optical resonances matches the absorption onset of the a-TiO_2_ active layer. We have hypothesized that such an optical design produces an enhancement of the electric field amplitude of the standing waves set up in the microcavity for wavelengths around the resonant feature and that this enhancement appears located at the center of the microcavity, i.e., at the a-TiO_2_ layer. Similar local enhancements of light electric field amplitude for given wavelengths are known to stem from interference processes occurring in a large variety of photonic structures including 1D Bragg microcavities.^29,51−53^

**Figure 5:**
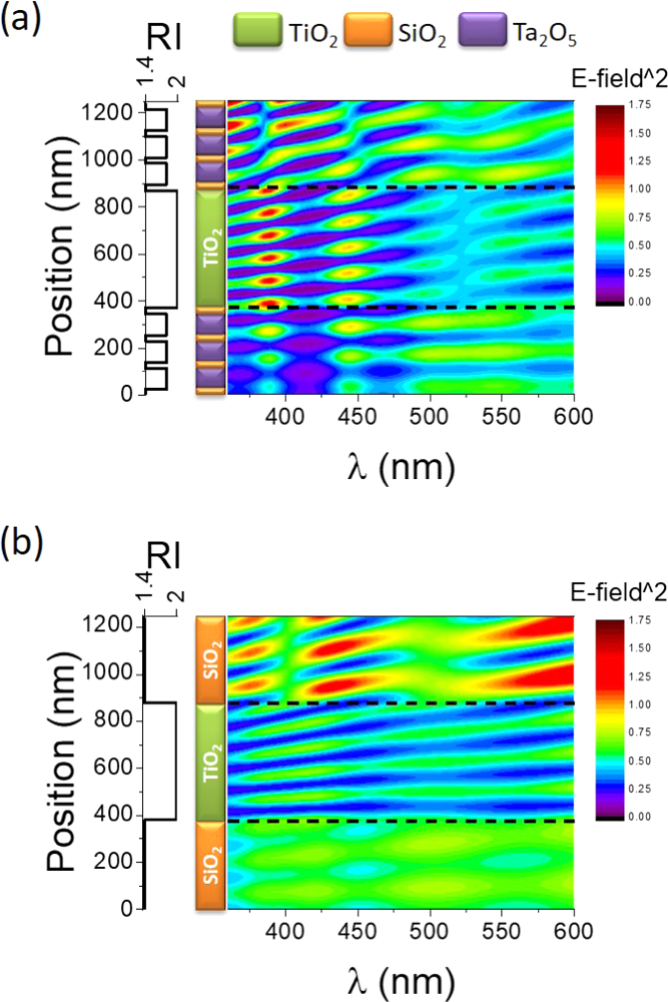
Multilayer stack schemes and electric field amplitude squared maps of the standing waves set up at BM/pA/BM (a) and S/pA/S (b) water-infiltrated samples under normal illumination. Horizontal dashed lines indicate the limits of the active a-TiO_2_ layer within the photonic structures. RI in-depth profiles are also indicated.

This hypothesis is supported by calculations carried out to map the spatial distribution of the electric field amplitude of the standing waves within the microcavity (see the Materials and Methods Section for details). Figure 5 shows, in the form of color maps, the electric field amplitude squared distribution as a function of the wavelength (*x*-axis) and local position within the layered structure (*y*-axis).^45^ The electrical field maps illustrate the developed standing waves for S/pA/S and BM/pA/BM water-infiltrated samples under normal illumination.

For the S/pA/S structure, there is a small enhancement of the electric field amplitude at the SiO_2_ upper layer for all wavelengths, while no particular enhancement effect is induced at the central a-TiO_2_ layer. This contrasts with the calculated electric field intensity distribution in sample BM/pA/BM, where, as evidenced by the development of six maxima in the color map (red spots), the electric field amplitude at wavelengths around 380 nm is strongly enhanced at the a-TiO_2_ central layer position. A significant though lesser enhancement is also found at around 440 nm (yellow spots). The number of maxima in this diagram depends on the number of complete half-waves that occupy the central cavity, since the condition to be fulfilled is that the wavelength of the resonance is a half-integer of the optical path of the cavity. In our case, it is remarkable that the special design of the BM/pA/BM photonic structure makes that this resonant condition holds for wavelengths around 385 and 440 nm for this sample immersed in water.

Note that light trapping is not related to the crystallographic phase of the stacked layers but to the spatial distribution of their refractive index. Thus, similar results are expected if the rutile titania active phase is considered as the central layer of the microcavity. However, one has to take into account that to get rutile samples, the multilayer system should be annealed at high temperatures,^3,4^ and this would most probably decrease the porosity of the films.

### 3.3 Enhanced Photocatalytic Degradation in 1D Resonant Microcavities

**Figure 6:**
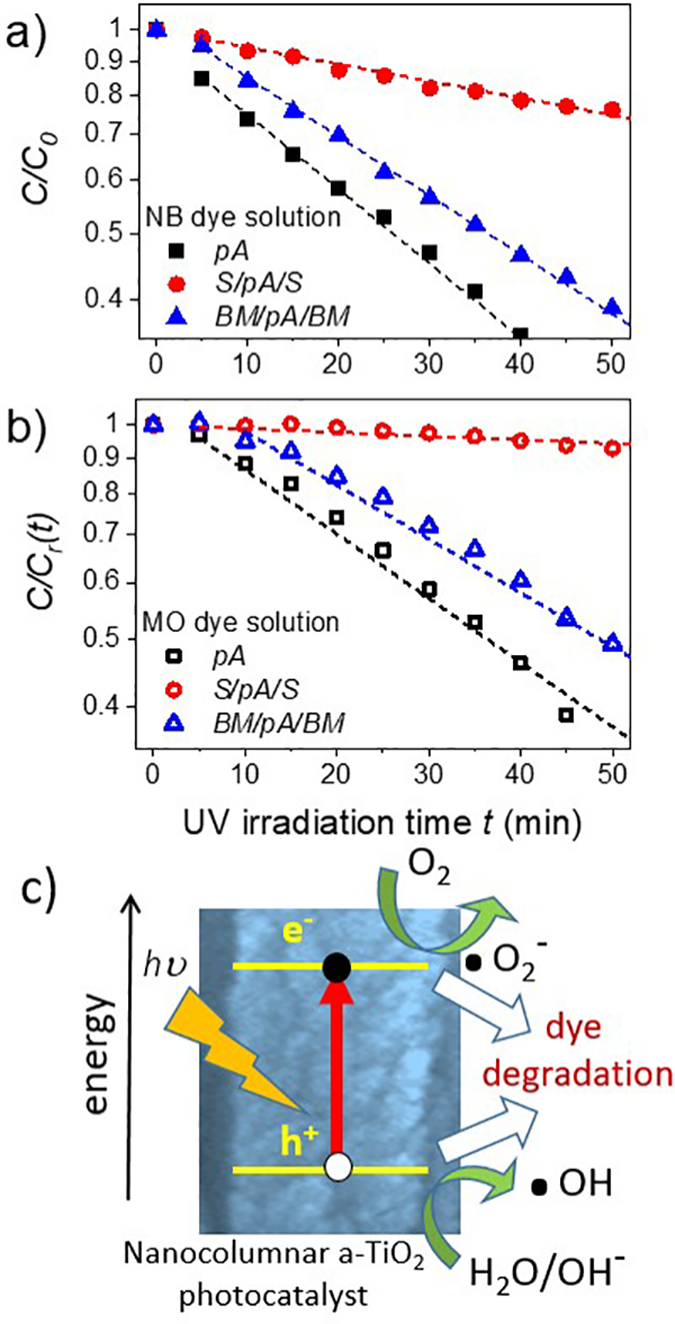
Photodegradation kinetics of the NB (a) and MO (b) dye solutions as a function of irradiation time of the layered systems containing the nanoporous a-TiO_2_ films that are investigated in this work. The straight lines correspond to linear regression fits to a pseudo-first-order kinetic model. (c) Schematic showing the typical light excitation and subsequent interface reactions of photogenerated electrons and holes at the columnar a-TiO_2_ nanostructures.^56^

Photocatalytic degradation tests of NB and MO dye solutions were carried out as reported in the Materials and Methods Section. Upon recording the absorbance spectra of dye solutions as a function of irradiation time with the Xe lamp, a progressive decrease in the intensity of the characteristic absorption bands at, respectively, 268 and 466 nm, was detected (cf., Figure S2 in the SI). From the intensity of these bands and its equivalence in terms of relative dye molar concentrations, it is possible to follow the kinetics of NB and MO photodegradation processes. Results are shown in Figure 6 where values of the relative dye concentrations vs UV irradiation time are plotted in a semilogarithmic scale.

The series of curves in this figure clearly shows that the two dyes become progressively degraded by the photocatalytic action of anatase. However, despite that the nanoporous a-TiO_2_ active layer thickness is similar in the three samples, the kinetics of the process strongly differ depending on the analyzed system. A first look at the curves in Figure 6a,b reveals that the photodegradation rate is much slower for sample S/pA/S, where the a-TiO_2_ layer is sandwiched between photocatalytically inactive porous capping films of SiO_2_. In general, these photodegradation processes may involve various controlling steps, including diffusion of reactants, photocatalytic oxidation, and so on. An approximation to make a semiquantitative comparison of photoactivities is to consider that the overall process can be described by a pseudo-first-order kinetics. The use of pseudo-first-order kinetic constants for a semiquantitative estimate of the diffusion restrictions has been proposed in similar tests with other porous TiO_2_ systems.^54,55^ Pseudo-first-order kinetic constants characterizing the rate of the photocatalytic degradation of NB and MO dyes can be calculated from the slope of these semilogarithmic plots according to the classical equation for first-order kinetic reactions: ln[*C*/*C*_0_] = −*k*_NB_*t* for NB or ln[*C*/*C*_r_(*t*)] = −*k*_MO_*t* for MO. Calculated values of pseudo-first-order kinetic constants corresponding to each sample and dye solution are summarized in Table 1.

**Table 1:** Pseudo-First-Order Kinetic Constants Determined for the Photocatalytic Degradation Reactions of Nitrobenzene and Methyl Orange Dye Solutions

	kinetic constants (min^–1^)		
dye solution	pA	S/pA/S	BM/pA/BM
nitrobenzene (NB)	0.0250	0.0057	0.0200
methyl orange (MO)	0.0212	0.0014	0.0170

The smaller kinetic constant values for MO and samples S/pA/S and BM/pA/BM in Table 1, in comparison with those of NB, point to higher diffusion constraints for MO than for NB dye, in agreement with its larger molecular size. Besides, the much smaller value of the photodegradation rates determined for sample S/pA/S with respect to that of the pA sample supports that in the sandwiched samples, the molecular diffusion of the dye, occurring through an approximately 600 nm thick porous layer, drastically reduces the activity of the central a-TiO_2_ layer. Remarkably, in sample BM/pA/BM, the pseudo-first-order kinetic constant approaches the values found for the bare pA film, even though diffusion restrictions similar to those in S/pA/S sample should also exist in this case. Therefore, we must conclude that additional factors must intervene in sample BM/pA/BM to compensate the loss of photocatalytic efficiency due to diffusion constraints. In other words, the measured degradation kinetics suggests an enhancement in the photocatalytic activity of the a-TiO_2_ active layer in sample BM/pA/BM. We assume that this enhancement is due to light trapping phenomena at the a-TiO_2_ layer for wavelengths around those of the optical resonances of this 1D Bragg microcavity (cf., Figures 3 and 4). In particular, we claim that the enhancement of the electric field intensity at the a-TiO_2_ layer in this 1D resonant microcavity is responsible for the observed boost in photocatalytic activity of this system with respect to sample S/pA/S.

The scheme in Figure 6c represents the classical mechanism proposed to account for the photodegradation of dye or organic molecules by UV-illuminated a-TiO_2_.^56^ Basically, it involves several steps: (i) an electron transfer from the valence to the conduction band of a-TiO_2_ upon absorption of a suitable UV photon; (ii) the migration of the valence band hole to the surface, where it is captured by an OH^–^ group to render reactive OH* radicals; (iii) the electron diffusion through the conduction band toward the surface and its reaction with oxygen to yield O_2_^–^ or similar species; and (iv) the subsequent reaction of the reactive species with the organic molecules initiating their degradation process. What we claim is that step (i) is more effective in sample BM/pA/BM due to the light trapping (i.e., local electric field enhancement) at the a-TiO_2_ layer predicted by the calculations in Figure 5.

### 3.4 Dye Giant Absorption at Resonant Microcavities

The electric field amplitude map in Figure 5b reveals the spatial distribution of two optical resonances at around 385 and 445 nm, both within the spread of the absorption band of MO and localized at the active a-TiO_2_ layer. In a previous publication where Rhodamine 101 was infiltrated within a similar porous 1D Bragg microcavity, we demonstrated that the absorption coefficient of the infiltrated dye strongly increases due to the enhancement of the local electric field of the standing waves set up at the central layer of the microcavity. Herein, we have verified whether a similar “giant” absorption mechanism may affect the absorption coefficient of the infiltrated MO molecules. For this purpose, we first evaluate the refractive index and absorption coefficient of the MO dye solution used in the photodegradation experiment. In the Supporting Information, we report the experimental transmittance spectrum for a 1 cm path cuvette filled with a 1.7 × 10^–5^ M MO dye solution, together with its fitting analysis and the retrieved optical parameters characterizing this aqueous dye solution (i.e., refractive index and absorption coefficient wavelength dispersion curves). According to the analysis in Supporting Information Figure S5, the absorption coefficient of the 1.7 × 10^–5^ M aqueous MO solution is ∼0.4 and ∼0.6 cm^–1^ at about 380 and 450 nm, respectively. Thus, the corresponding molar absorption coefficients (α_Mmax_) at these wavelengths are 2.3 × 10^4^ and 3.5 × 10^4^ cm^–1^ M^–1^, respectively.

**Figure 7:**
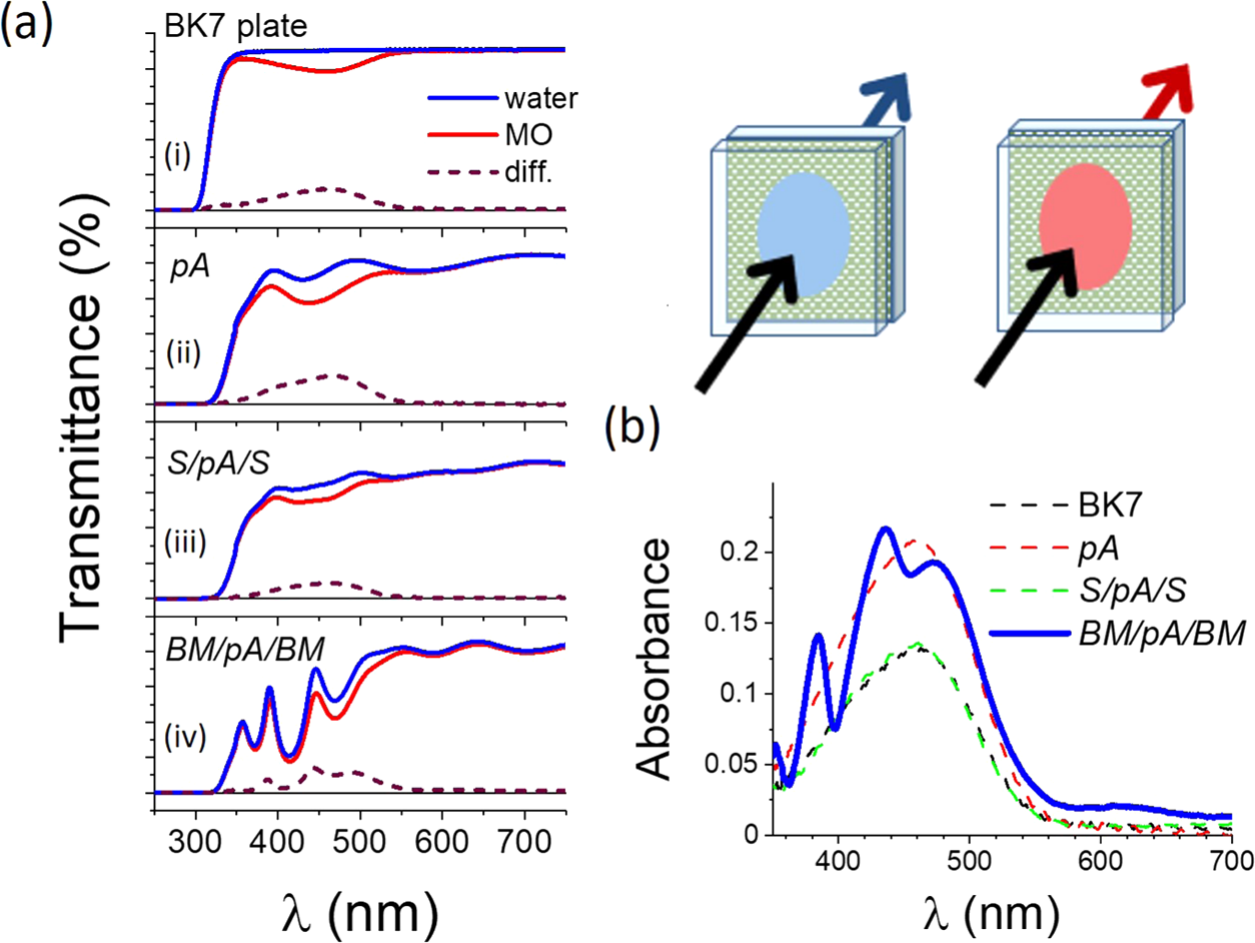
(a) Transmittance spectra of a film of water (blue lines) or a MO dye solution (red lines) trapped by capillary forces between a BK7 glass plate and (i) another BK7 plate, (ii) sample pA, (iii) sample S/pA/S, and (iv) sample BM/pA/BM; these samples deposited on fused silica plates. The dashed lines are the corresponding difference spectra. (b) Absorbance of the trapped liquid in the four studied cases. The scheme shows the configuration of plates adopted for the experiments.

In addition, we measured the transmittance of a liquid film of either water or concentrated MO dye solution (1.7 × 10^–3^ M) trapped by capillarity forces between a BK7 glass plate and: (i) another BK7 glass plate, (ii) sample pA, (iii) sample S/pA/S, and (iv) sample BM/pA/BM. In all cases, the layered structures were deposited on fused silica plates. It is noteworthy that in this experiment, a strict control on the thickness of the liquid layer trapped between the two plates is not possible and that therefore the absolute intensity of the absorption curves is not relevant for the analysis. Figure 7a shows that the shape of the interference fringes of the transmittance spectra obtained in each case is only slightly modified when changing water for the MO 1.7 × 10^–3^ M aqueous solution, indicating that both liquids have very similar refractive indices (in fact, their RI differ by less than 0.001 RIU).

Nevertheless, a certain modification in the transmittance curves observed between 350 and 550 nm can be associated with the absorption of the MO dye. In fact, the ratio in Figure 7b between the curves recorded with water and the dye solution (i.e, equivalent to the absorbance of the trapped dye solution in each case) for the studied cases (i)–(iii) renders spectral curves with a similar wavelength dependence to the absorption of MO dye solutions measured in a cuvette (cf. Figure S5). Thus, for these cases, a rough estimation of the thickness of the dye solution layer *t*_dye_ can be obtained with the expression
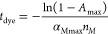
1where *A*_max_ and α_Mmax_ are the absorbance and molar absorption coefficient at the wavelength of maximum absorption of the dye solution (i.e., ∼450 nm), and *n*_M_ its molar concentration. According to the previous simple expression, the liquid thickness *t*_dye_ trapped by capillary forces between the plates in cases (i), (ii), and (iii) varies between 2 and 4 μm. This 100% variability stands for the already mentioned lack of control in this experiment over the thickness of the liquid layer trapped between the glass plate and the fused silica-supported multilayers.

However, remarkably, the absorbance in Figure 7b corresponding to the concentrated MO dye solution sandwiched between a glass plate and BM/pA/BM porous microcavity deposited on fused silica shows that, superimposed to the absorption of the free dye solution, there appear a series of discrete peaks that match the optical resonances of the Bragg microcavity. Considering the molar absorption coefficients for the dye solution determined above (i.e., α_Mmax_ equal to 2.3 × 10^4^ and 3.5 × 10^4^ cm^–1^ M^–1^ at 380 and 450 nm, respectively), a rough estimation of the absorbance of a given MO dye solution with an equivalent thickness *t*_void_ (i.e., reproducing the volume of the void fraction available in the porous pA film (i.e., *t*_void_ = ∼200 nm)) can be evaluated according to the following expression
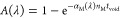
2

According to this equation, the expected absorbances of a 200 nm thick layer of 1.7 × 10^–3^ M MO dye solution evaluated at the optical resonances of the microcavity, i.e., at 380 and 450 nm, are 0.008 and 0.012, respectively. In other words, in the absence of light trapping phenomena, the MO dye absorption of the solution infiltrated within the microcavity would have a negligible intensity and hardly any specific feature would be detected superimposed onto the absorption of the solution layer trapped between the glass and the BM/PA/BM sample. We assume that, according to previous studies, the significantly higher experimental absorbances (about 1 order of magnitude higher) depicted in Figure 7b at these wavelengths must be due to a “giant absorption” effect. Following previous works,^57,58^ this enhancement of absorption coefficient is proportional to the local electric field amplitude squared times the local effective refractive index in the Bragg microcavity. Thus, the spectrum in Figure 7b shows that, at the wavelengths of the optical resonances, the absorption coefficient intensity of the dye fraction infiltrated in the nanoporous a-TiO_2_ central layer in the Bragg microcavity is about 10 times stronger than in the free solution. An enhancement of the same order of magnitude has been reported for the absorption of Rhodamine 101 ethanol solutions infiltrated in similar porous 1D Bragg microcavities.^28^

### 3.5 Visible-Light-Induced Photodegradation at Resonant 1D Microcavities

**Figure 8:**
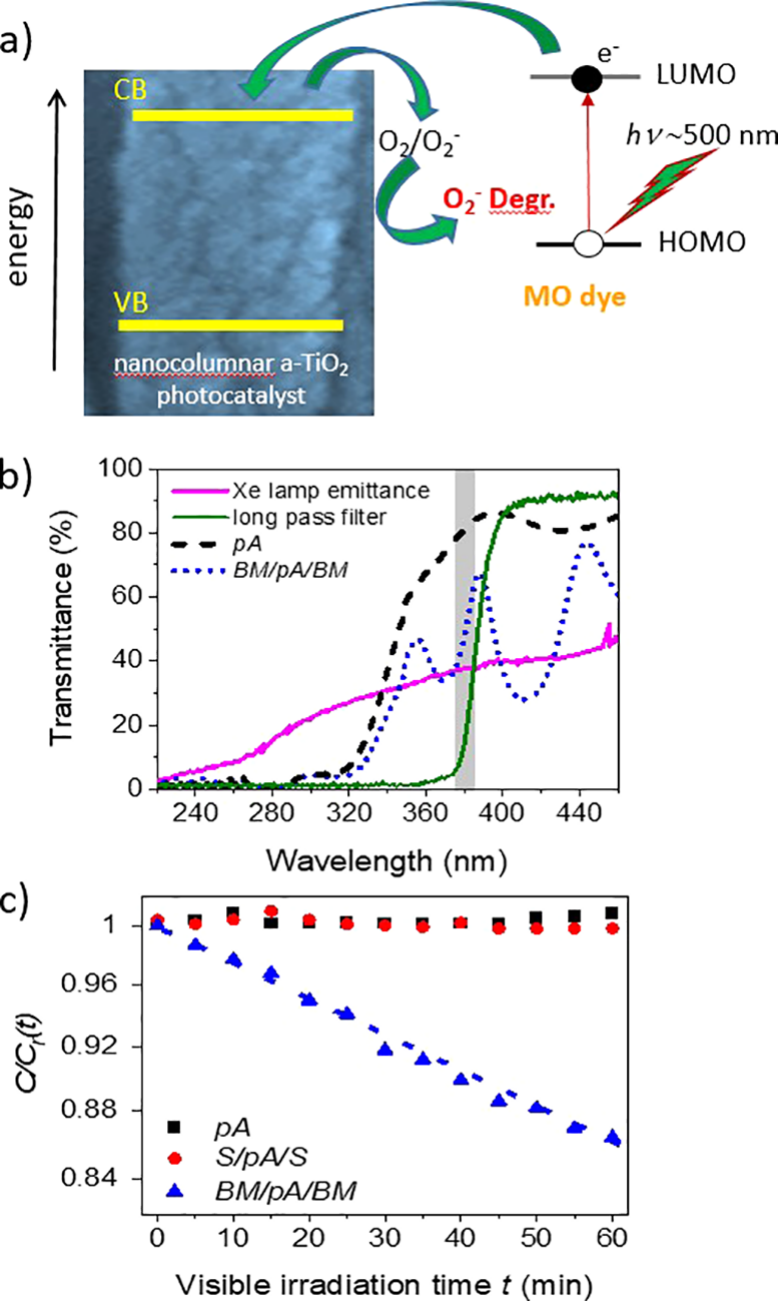
(a) Visible-light-induced photodegradation mechanism: (i) photoexcitation with visible light inducing a transition from the ground to an excited state of the dye molecule; (ii) electron transfer from the dye excited state to the a-TiO_2_ conduction band; (iii) ensuing photodegradation through direct reaction of the dye excited state with water and/or dye molecules with O_2_^–^ species produced by the reaction of the transferred electron with oxygen in the medium. (b) Transmittance of pA and BM/pA/BM samples compared with the Xe lamp emittance and the transmittance of the long-pass filter utilized for these experiments. (c) Visible-light-induced MO dye photodegradation kinetics upon irradiation through a long-pass filter with a cutoff wavelength of ∼385 nm (note that the *y*-scale is different than in the plots in Figure 6).

In general, visible light is inefficient to induce any photocatalytic reaction in a-TiO_2_ because it cannot generate electron–hole pairs at the surface of this semiconductor and, therefore, cannot trigger any degradation process.^1,3^ Nevertheless, under certain circumstances, it has been claimed that dye degradation may occur with visible light if an electron transfer occurs from an excited state of the dye to the conduction band of the a-TiO_2_.^19^ This process is similar to that occurring in dye-sensitized photovoltaic cells, although in that case, the electron transfer from the dye to the conduction band and the resulting formation of an ionized molecule is reversed by the reaction of the latter with iodine ions present in the medium.^59,60^ A band scheme of this visible-light-induced dye photodegradation process is shown in Figure 8a. In practice, under standard experimental conditions with powder or simple thin-film samples, this mechanism is quantitatively negligible, but it has been claimed to occur in 3D photonic crystal structures where light confinement effects may produce an exaltation of the absorption coefficient of the dye.^19^

Herein, we have investigated whether visible light might induce the photodegradation of MO dye upon irradiation of a-TiO_2_ thin film samples to induce the photosensitization of the semiconductor by visible light excitation of the dye molecules. For this experiment, we used the same Xe lamp as in the previous photodegradation experiments, but placed a long-pass optical filter (cutoff wavelength ∼385 nm), consisting of a PMMA plate, between the lamp and the cuvette, where the degradation experiments are carried out. For a better assessment, in Figure 8b, we compare the transmittance of this filter with the spectrum of the light emitted by the Xe lamp together with the transmittance of pA and BM/pA/BM samples deposited on fused silica plates. According to these spectra, the insertion of this long-pass filter removes most UV photons for wavelengths shorter than 385 nm, thus making unlikely any photodegradation process based on the classical photoexcitation mechanism of titania consisting of the generation of electron–hole pairs at, respectively, its conduction and valence bands and their reaction with the dye molecules (cf. Figure 6c).^1,3^ The absence of any photodegradation was, in fact, found for NB dye solutions with its absorption maximum at 290 nm in all investigated samples (data not shown). Unlike NB dye, MO dye presents its maximum absorption at 466 nm with a band extending from 350 to 550 nm (see Supporting Information Figure S2), which, according to the analysis summarized in Figure 7, overlaps the optical resonance features of the BM/pA/BM microcavity, particularly that around 440 eV.

According to Figure 8c, within the experimental error, no measurable visible-light-induced photodegradation of MO dye was observed with pA and S/pA/S samples. However, a certain photodegradation was measured with sample BM/pA/BM. After data evaluation, a pseudo-first-order kinetic constant of 0.0025 min^–1^ was obtained for these experimental conditions. We propose that this little but not negligible degradation occurs through a process of the kind depicted in Figure 8a, involving the following steps:^19^ (i) the visible light excitation of the MO molecule to a MO* state through a HOMO–LUMO transition; (ii) the electron transfer from the excited state of the molecule MO* to the conduction band of the TiO_2_ semiconductor and the formation of an ionized MO^+^ molecule (i.e., the dye photosensitization of the a-TiO_2_); (iii) the reaction of the conduction band electron with oxygen and the formation of very reactive O_2_^–^ or similar species; and (iv) the reaction of MO^+^ with water or O_2_ and/or O_2_^–^ and derived species of oxygen.

Therefore, we attribute the photodegradation of the MO dye upon visible light irradiation of the BM/pA/BM microcavity reported in Figure 8c to the enhancement of the light absorption process by the dye molecules in the solution infiltrated in the central a-TiO_2_ nanoporous layer of the microcavity, as illustrated by the analysis in Figure 8. In the other samples, although electron transfer processes as those proposed in Figure 8a might also take place, their incidence would be very small and, in practice, undetectable under the operating conditions of our experiment.

## 4 Conclusions

We have proved that the photoactivity of a nanoporous a-TiO_2_ thin film is boosted when it is incorporated within a porous 1D optical microcavity. The enhancement of photocatalytic activity is due to light confinement effects for photons with wavelengths around the absorption onset of a-TiO_2_. Locally, this enhancement takes place at the central layer of the optical microresonator where the titania layer is located. We claim that this enhancement effectively compensates the deterioration in photoactivity observed when the a-TiO_2_ film is sandwiched between inactive porous layers imposing reactants/products diffusion constraints to reach the photocatalytically active layer.

Another evidence gained from the analysis of the activity upon visible light exposure of the 1D optical microcavity infiltrated with dye solutions is that an enhancement in the absorption coefficient of the dye promotes an additional degradation process. This involves both a “giant absorption” mechanism of the dye molecules infiltrated in the porous a-TiO_2_ layer and their ionization through an electron transfer to the conduction band of the semiconductor.

The reported experimental evidence and simulation analysis of the optical microcavity incorporating a nanoporous active titania layer suggest that the engineering of the photonic response of porous 1D layered systems allows to enhance their photocatalytic activity both with UV and visible light, in this latter case promoting an additional activation mechanism of dye molecules.

Finally, it is noteworthy that light trapping phenomena of the same nature as those described in the present work may be designed for other 1D photonic structures (e.g., making that light trapping occurs at the outer layers of the photonic structure and/or other layer stack distribution). We believe that through proper engineering of the photonic response, it would be possible to combine the enhancement of photoactivity reported in this work with the incorporation of additional functionalities (e.g., structural color, sensing capacity as response to their infiltration with liquids of different refractive indices and other). This constitutes another asset to be considered for the future manufacturing of this type of nanoporous multifunctional photonic structures.

## Supporting Information Available

Experimental setup for the photodegradation kinetics studies; evaluation of photodegradation kinetics; structural analysis of the a-TiO_2_ layered samples; fitting analysis of the transmittance spectrum of Bragg microcavities; and optical analysis of the MO dye solutions (PDF)
