# Supplementary material for: Titania Enhanced Photocatalysis and Dye Giant Absorption in Nanoporous 1D Bragg Microcavities
Source: ACS Appl Nano Mater. 2022 Apr 7;5(4):5487–97. doi: 10.1021/acsanm.2c00477 (PMC9040112; doi:10.1021/acsanm.2c00477)
Supplement: Supplementary file 1 — an2c00477_si_001.pdf [file an2c00477_si_001.pdf]

Supporting information

Titania Enhanced Photocatalysis and Dye Giant Absorption in Nanoporous 1D Bragg Microcavities

Victor J. Rico*, Halime Turk, Francisco Yubero*, Agustin R. Gonzalez-Elipe**

Instituto de Ciencia de Materiales de Sevilla (CSIC-Univ. Sevilla). Avda. Américo Vespucio 49. E-41092 Sevilla. Spain.

**Corresponding authors:* victor@icmse.csic.es, yubero@icmse.csic.es

***On leave from Faculty of Physics, Engineering, Earth, Environment, Mechanics. University Grenoble Alpes. 126 Rue de la Physique, 38400 Saint-Martin-d'Hères 38040 Grenoble. France.*

Supporting information S1.

Experimental set-up used for the dye photodegradation kinetics studies

According to **Figure S1**, a fused silica cuvette (2x1x4 cm^3) (1) was filled with 4 cc of dye solution. The photoactive samples (2) (2x2 cm^2) deposited on polished silicon wafer substrates were immersed in the dye solution. Oxygen was continuously bubbling through the dye solution (3) to ensure that photodegradation kinetics was not limited by any shortage of this reactant. A Teflon cap (4) prevented that liquid level decreased because the oxygen bubbles dragged part of the liquid during the photodegradation experiment. Degradation tests were carried out irradiating with a Xe lamp (LASING ASB-Xe-175) (5) located at 15 cm from the cuvette, with an irradiance of 1.8 W cm^{-2} at the position of the samples for the complete UV + Vis spectrum (ca. 0.3 W cm^{-2} corresponded to photons with wavelength shorter than 380 nm). The kinetics of the photodegradation process was monitored following the time evolution of the maximum of the absorption bands of nitrobenzene (NB) and methyl-orange dyes (MO) at, respectively, 268 and 466 nm. The absorbance spectra were recorded illuminating with a halogen lamp (6), carrying the light with an optical fiber (7) to one of the windows of the cuvette, and collecting the transmitted light at the opposite window (7), transversally to the irradiation of the Xe lamp used to activate the photodegradation of the dye solutions. An Ocean Optics MAYA 2000-pro UV-Vis

spectrometer (8) was used to collect absorbance spectra every 5 min during two hours. **Figure S1** shows a scheme of this experimental set-up.

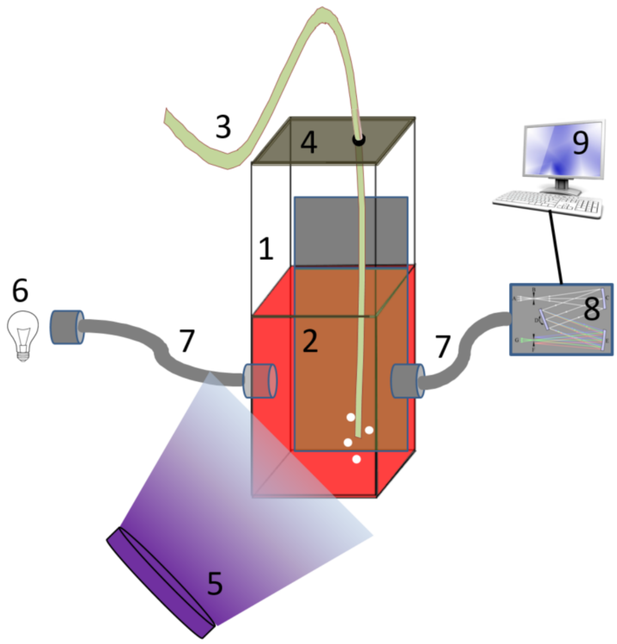

Figure S1. *Experimental set-up for evaluation of the dye photodegradation kinetics: (1) fused silica cuvette, (2) dye solution, (3) oxygen supply, (4) Teflon cap, (5) Xe lamp used to activate dye photodegradation, (6) halogen lamp used to perform absorption measurements, (7) optical fibers, (6) oxygen supply, (8) monochromator, (9) laptop*

Supporting information S2.

Evaluation of photodegradation kinetics

In the analysis of the photodegradation kinetics of the dye solutions induced by the TiO_2 photocatalyst material (i.e., either in *pA, S/pA/S or BM/pA/BM* samples), it is assumed that the dye concentration in the solution is proportional to their absorbance.

It is found that in the absence of the photocatalyst agent (i.e., the a-TiO_2 based samples), the NB dye solution is stable upon irradiation with the UV Xe lamp, while this is not the case with the MO dye solution, that underwent a little but progressive degradation upon UV irradiation.

To account for the dye degradation induced by direct UV light exposure in the absence of the photocatalyst agent, the dye concentration data are reported normalized, i.e., in the form of C/C_0 for the NB solution (with C_0 the concentration of NB at time t=0) or $C/C_r(t)$ for the MO solution (with $C_r(t)$ the concentration of the MO at time t upon

exposure to the Xe lamp without photocatalytic sample in the solution). Two selected examples of the evolution of the absorption bands of NB and MO during photodegradation experiments under Xe-lamp irradiation in the presence of the *pA* photocatalyst sample are shown in **Figure S2.** The intensities at the maxima of the corresponding absorption bands at 268 and 466 nm are used to characterize the photodegradation kinetics of the different porous a-TiO_2 containing samples.

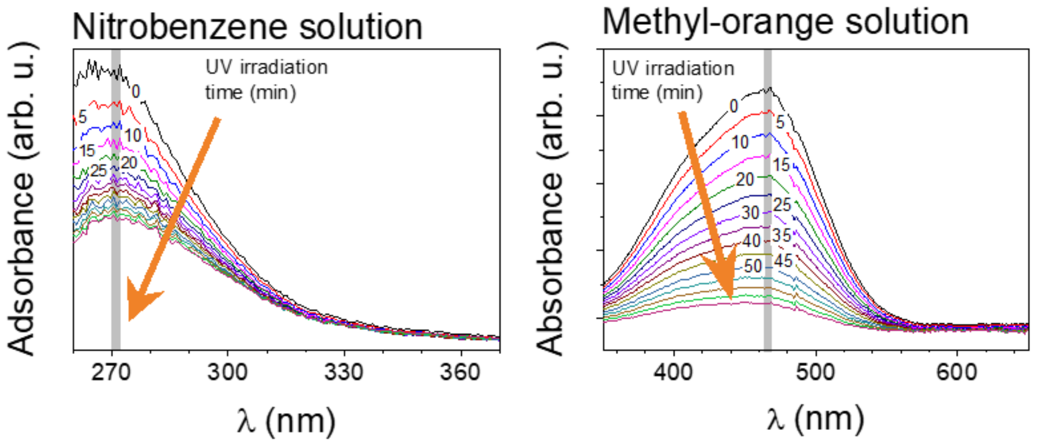

Figure S2. *Absorbance corresponding to the kinetic degradation experiments of NB and MO solutions under UV irradiation with the active pA sample*

Supporting information S3.

Structural analysis of the a-TiO_2 layered samples

Structural analysis was done by means of X-ray diffraction (XRD) measurements carried out in a Siemens D5000 diffractometer system, employing monochromatic Cu-Kα radiation, 0.02° step angle and 60 s measuring time per step. All the samples were annealed at 400 ºC in air during 3 hours to induce the crystallization of the active anatase phase of titania. **Figure S3** shows the diffractograms corresponding to the *pA, S/pA/S, and BM/pA/BM* samples. The main diffraction peaks of anatase titania are indicated in the figure.

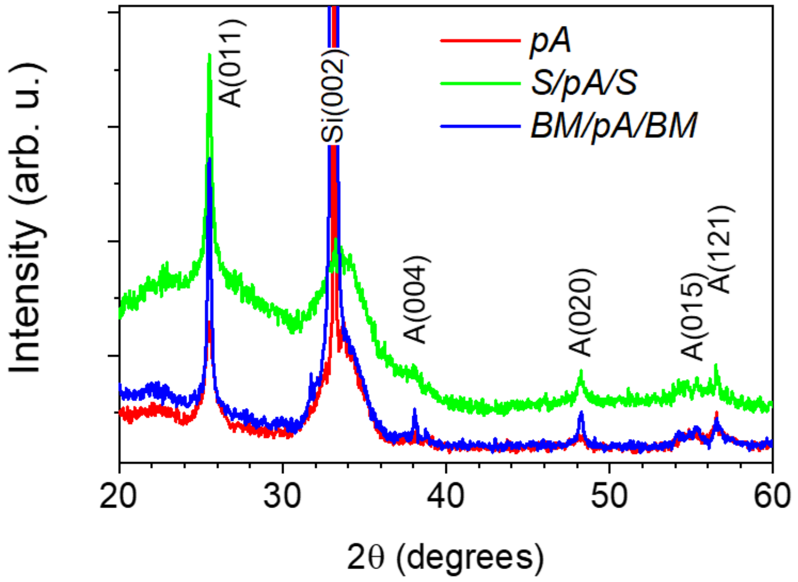

Figure S3. XRD diffractograms corresponding to pA, S/pA/S, and BM/pA/BM samples

Supporting information S4.

Fitting analysis of the transmittance spectrum of Bragg microcavities

Transmittance spectra of the resonant microcavity were recorded in a UV/VIS/NIR PerkinElmer Spectrometer (Lambda 750S) for samples deposited on fused silica substrates, either in their pristine state (i.e., empty) or immersed a 1 cm width cuvette filled with water. These spectra were modeled by the transfer matrix method with the WVASE® software (J. A. Woollan Co). The parameters obtained from the fitting procedure (thicknesses and refractive indices of all the layers within the multilayer stack) were used to evaluate the spatial distribution of the electric field amplitude vs wavelength, corresponding to the light confinement effects developed in the microcavity.

Due to the high number of variables to be considered in the fitting analysis (15 layers with the parameters defining their refractive indices, extinctions coefficients, void fractions and thicknesses), several constrains were considered:

- All layers of the same material were modeled with the same refractive index and extinction coefficient

- Identical void fraction was considered for all the layers of the microcavity. Porosity was modeled assuming a Bruggeman effective medium approximation.
- Independent fine tuning of thickness for each layer within the stack was allowed to optimize the fitting of the interference fringes in the transmittance spectra.
- The microcavity sample was simulated with the same material parameters (refractive, indices, extinction coefficients, void fraction) and layered stack structure, independently of the media filling the pore structure (either air or water).

With these constraints, the detailed stack structure and material parameters obtained are included in **Tables S1** and **S2**:

Table S1. Detailed multilayer stack structure. L and H refer to low and high refractive index material within the capping Bragg mirror. The numbers before L and H indicate the thickness (in nm) of the corresponding film

Sample ID	Stack structure
BM/pA/BM	$(25L87H)^3$25L/495pA/$(25L87H)^3$25L

Table S2. Refractive indices (RI) evaluated at 450 nm and pore volume fraction obtained from fitting analysis

Material	RI @450nm (pristine, empty)	RI @450nm (water infiltrated)	Pore volume fraction
Porous SiO_2 (L)	1.26	1.38	40%
Porous Ta_2O_5 (H)	1.66	1.81	40%
Porous TiO_2 (pA)	1.83	1.93	40%

Wavelength and in-depth electric field amplitude distribution maps within the microcavities were evaluated with FILMSTAR Optical Thin Film software. The inputs for this calculation were the optical constants (i.e., refractive index and extinction coefficient of the layers in the microcavity) and the stack structure of the microcavities

obtained from the WVASE analysis of the measured transmittance spectra and the cross sectional SEM images.

Supporting information S5.

Optical analysis of the MO dye solutions

The optical properties (refractive index, extinction coefficient) of the absorbing aqueous dye solutions were described through a parametrized GENOSC.MAT material model included WVASE32 software (J.A. Woollan Co.). It consisted on the superposition of a Cauchy wavelength dispersion corresponding to the water solvent, plus the Gaussian-Lorentzian absorption bands resulting from the fitting of the experimental transmittance of the resonant microcavities. The model couples self-consistently the refractive index and extinction coefficient of the dye solution through Kramers−Kronig relations. **Figure S5a** shows the experimental transmittance of a 1.7x10^{-5} M aqueous MO solution in a 10 mm path cuvette together with its fitting analysis and the retrieved optical parameters characterizing the dye solution. Refractive index and absorption coefficient wavelength dispersion curves are reported in **Figure S5b**.

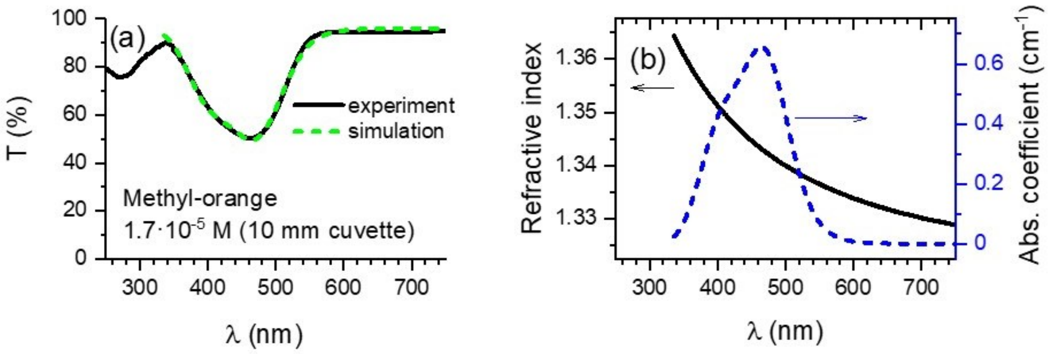

Figure S5. (a) Transmittance spectra of a 1.7 10^{-5} M aqueous MO solution through a 10 mm cuvette (full line) and simulated spectrum (dashed line). (b) Refractive index and absorption coefficient corresponding to the dye solution.
